# Effectiveness of 32 versus 20 weeks of prednisolone in leprosy patients with recent nerve function impairment: A randomized controlled trial

**DOI:** 10.1371/journal.pntd.0005952

**Published:** 2017-10-04

**Authors:** Inge Wagenaar, Erik Post, Wim Brandsma, Bob Bowers, Khorshed Alam, Vanaja Shetty, Vivek Pai, Sajid Husain, Cita Rosita Sigit Prakoeswa, Linda Astari, Deanna Hagge, Mahesh Shah, Kapil Neupane, Krishna Bahadur Tamang, The TENLEP study group, Peter Nicholls, Jan Hendrik Richardus

**Affiliations:** 1 Dept. of Public Health, Erasmus MC, University Medical Center Rotterdam, Rotterdam, the Netherlands; 2 KIT Health, Royal Tropical Institute, Amsterdam, the Netherlands; 3 Independent Leprosy Consultant, Royal Tropical Institute, Amsterdam, the Netherlands; 4 Rural Health Program, The Leprosy Mission International Bangladesh, Nilphamari, Bangladesh; 5 Menzies Health Institute Queensland, Griffith University, Brisbane, Australia; 6 Foundation for Medical Research, Mumbai, India; 7 Bombay Leprosy Project, Mumbai, India; 8 JALMA institute of Leprosy & Other Mycobacterial Diseases, Agra, India; 9 Dermatovenereology Dept., Dr Soetomo Hospital–Faculty of Medicine, Universitas Airlangga, Surabaya, Indonesia; 10 Anandaban Hospital and Mycobacterial Research Laboratories, The Leprosy Mission Nepal, Kathmandu, Nepal; 11 Lalgadh Leprosy Hospital and Services Centre Dhanusha, Lalgadh, Nepal; 12 Faculty of Health Sciences, University of Southampton, Southampton, United Kingdom; University of California San Diego School of Medicine, UNITED STATES

## Abstract

**Background:**

While prednisolone is commonly used to treat recent nerve function impairment (NFI) in leprosy patients, the optimal treatment duration has not yet been established. In this “Treatment of Early Neuropathy in Leprosy” (TENLEP) trial, we evaluated whether a 32-week prednisolone course is more effective than a 20-week course in restoring and improving nerve function.

**Methods:**

In this multi-centre, triple-blind, randomized controlled trial, leprosy patients who had recently developed clinical NFI (<6 months) were allocated to a prednisolone treatment regimen of either 20 weeks or 32 weeks. Prednisolone was started at either 45 or 60 mg/day, depending on the patient’s body weight, and was then tapered. Throughout follow up, NFI was assessed by voluntary muscle testing and monofilament testing. The primary outcome was the proportion of patients with improved or restored nerve function at week 78. As secondary outcomes, we analysed improvements between baseline and week 78 on the Reaction Severity Scale, the SALSA Scale and the Participation Scale. Serious Adverse Events and the need for additional prednisolone treatment were monitored and reported.

**Results:**

We included 868 patients in the study, 429 in the 20-week arm and 439 in the 32-week arm. At 78 weeks, the proportion of patients with improved or restored nerve function did not differ significantly between the groups: 78.1% in the 20-week arm and 77.5% in the 32-week arm (p = 0.821). Nor were there any differences in secondary outcomes, except for a significant higher proportion of Serious Adverse Events in the longer treatment arm.

**Conclusion:**

In our study, a 20-week course of prednisolone was as effective as a 32-week course in improving and restoring recent clinical NFI in leprosy patients. Twenty weeks is therefore the preferred initial treatment duration for leprosy neuropathy, after which likely only a minority of patients require further individualized treatment.

## Author summary

Nerve damage is a common and severe consequence of leprosy, and it can lead to impairment of sensory (sensibility) or motor (muscle) function of hand, feet and eyes. To prevent that the nerve damage becomes permanent, it is essential that new damage is treated in an early stage. Prednisolone, an immune suppressor, is the drug of choice. However, the ideal treatment duration is unknown. In our study, we compared a prednisolone treatment of 20 weeks with a longer treatment of 32 weeks. After the follow up period of 1.5 years, the percentage of patients with an improved or completely restored nerve function was the same in the two treatment groups, namely 78%. We can conclude that the longer treatment duration was not more effective, and that 20 weeks of prednisolone is sufficient to treat early nerve damage in most patients. We also found that in 15% of the patients neither one of the treatments was effective. For this specific group, more research needs to be done to find if alternative medication or perhaps even longer prednisolone treatment can be helpful.

## Introduction

Leprosy is an infectious disease caused by *Mycobacterium leprae*. Since the introduction of antibiotic multidrug treatment (MDT) in the 1980’s, the number of leprosy diagnoses has decreased dramatically, and the disease was even declared eliminated as a public health problem at a global level in the year 2000 –i.e. less than 1 case per 10 000 inhabitants. Nevertheless, in 2015 a total of 210 000 new leprosy patients were diagnosed worldwide [1].

A main complication of leprosy is neuropathy, which often causes sensory and motor nerve function impairment (NFI). Untreated NFI can result in deformities of the hands and feet and may also affect the eyes. NFI can develop before MDT has started, but it can also arise during MDT and even several years after leprosy treatment has been completed [2,3]. The risk of developing new NFI within two years of starting MDT can be as high as 65% [4].

To prevent disabilities and deformities in leprosy patients, it is very important to detect and treat neuropathy in an early stage. Neuropathy is commonly treated with prednisolone [5], an immune suppressor that reduces the body’s immune responses towards *M*. *leprae* and relieves the pressure on the nerves by reducing inflammation and oedema [6,7].

Although ideally prednisolone therapy is adjusted to individual needs and response, this is not always feasible in field clinics, which often lack the treatment expertise of referral centres [8]. In these situations, the WHO recommends a standardized prednisolone treatment for 12 weeks [9].

Even though observational studies suggest that prednisolone can improve nerve function in 60–70% of nerves [2, 10–12], this effect has never been established in randomized controlled trials (RCT) [13]. There are indications, however, that a longer treatment duration may be more effective than the WHO standardized treatment. In an RCT in India, type 1 reaction (T1R) patients on a 20-week prednisolone course required less additional prednisolone than patients on a 12-week course [14]. Further research is needed to establish the optimal prednisolone regimen specifically for leprosy patients with NFI.

For this reason, we designed a study entitled “Treatment of Early Neuropathy in Leprosy” (TENLEP), comprising two RCTs aimed at determining how prednisolone treatment best can prevent permanent NFI. In this paper we describe the results of the Clinical trial, in which we evaluated whether a 32-week prednisolone course is more effective than a 20-week course in restoring and improving recent clinical neuropathy (<6 months) [15].

## Methods

The TENLEP study was a multicentre, triple blind parallel-group clinical trial, conducted in six referral centres for leprosy in India, Nepal, Bangladesh and Indonesia. A more detailed description of the TENLEP study can be found in the study protocol paper [15].

### Ethics statement

Before trial initiation, approvals were obtained in each country from the appropriate ethical review committees: in India from the Indian Council of Medical Research and the Ethics Committee of the Foundation for Medical Research, Mumbai (FMR/IEC/LEP.01b/2011); in Nepal from the Nepal Health Research Council (Reg.no 14/2011); in Indonesia from the Komite Etik Penelitian Kesehatan RSUD Dr. Soetomo Surabaya (100/Panke.KKE/V/2011); in Bangladesh from the Bangladesh Medical Research Council- National Research Ethics Committee (BMRC/NREC/2010-2013/533). Written informed consent was taken from all patients, or their parents in case the patient was under the age of 18 years. The trial was registered in the Netherlands Trial Register (NTR2300) and in the Clinical Trials Registry India (CTRI/2011/09/002022 and 23).

### Patients

Leprosy patients between 15 and 60 years of age with any recent peripheral NFI (less than 6 months onset) were eligible for the trial. Leprosy diagnosis and classification assignment was confirmed with physical exam by an experienced clinician, physiotherapist exam for neuropathy and slit skin smear microscopy. Ridley-Jopling classification was clinically assigned except in Anandaban hospital, where skin biopsy histopathology was additionally employed. NFI was established with voluntary muscle testing (VMT) and/or monofilament testing (MFT). Patients were excluded if they were pregnant, already receiving prednisolone treatment, suffered from other conditions that may affect the peripheral nervous system, or presented with a single skin lesion on the trunk as the only sign of leprosy. The sample size was calculated to be able to detect ‘restored or improved’ nerve function in 70% of the intervention group, compared to an assumed proportion of 60% in the control group. This one-tailed hypothesis, using 80% power, 5% significance and allowing for 20% loss to follow-up, lead to a sample size of 720 patients.

### Treatment

Prednisolone dose started at 45 mg/day for patients with low weight (≤50 kg) and at 60 mg/day for patients with high weight (>50 kg). The prednisolone dose was then slowly tapered during the treatment period, maintaining a plateau of 20–35 mg/day for 20 weeks in the 32-week arm–depending on weight group. Total dosage and dose over time are previously described [15]. To check the chemical composition of the prednisolone and placebo tablets, a random selection of packages from both treatment groups was evaluated at the start of the trial by the manufacturer (Rubicon), and by an independent Indian laboratory (Medibios Laboratories). After 20 months, the composition was checked again by the Royal Dutch Society of Pharmacists (KNMP). Treatment adherence was checked every month either verbally or by checking the medication package of the previous month.

### Randomization and blinding

Patients were randomly allocated to either 20 or 32 weeks of oral prednisolone, using a separate computer-generated blocked randomization sequence for each centre and weight group, blinded within pre-set patient numbered packaging. Patients were kept blinded, as the tablets appeared the same and treatment duration was kept equal by using placebo tablets. In addition, research staff and the statistician were kept blinded until all data analyses were performed. The key to treatment allocation was only broken if a patient had a serious adverse event (SAE) or required individualized treatment for a reaction or worsening NFI.

### Assessments

In each centre, monofilament testing and voluntary muscle testing were carried out by two trained assessors, except in the Indonesian centre where ten assessors have performed the tests. Assessments at baseline generally showed good inter-tester reliability [16]. For every patient, the sensory function of six nerves and the motor function of seven nerves were assessed on both left and right body side (total of 26 assessments). Sensory function was tested on three test-sites for each nerve, using a standard set of Semmes-Weinstein monofilaments, with the 200mg filament representing the normal threshold for the hand, and the 2g filament for the foot. For motor function assessment, the 0–5 Medical Research Council (MRC) scale was used [17]. The exact test methods and sites are previously described [13]. When the total monofilament score for a nerve was 3 or more, the sensory nerve function was considered impaired. A motor nerve scoring less than 5 on the Medical Research Council scale was also regarded as impaired. Follow-up assessments for VMT and MFT were carried out monthly during the treatment period (up to week 32), and at week 52 and 78. At baseline and the end of the study a Screening of Activity Limitation and Safety Awareness (SALSA) scale [18] and a Participation (P) scale [19] were completed for each patient. In addition, reaction severity was monitored with a Reaction Severity Scale (RSS) [20] at baseline, week 32, 52 and 78. When a patient did not show up for their follow-up appointment, a telephone call or in some cases a home visit was made in an effort to get the patient visit the clinic for assessments. When indicated in advance, a patient was allowed to miss one assessment, and the medication was provided for eight weeks.

As previously described, if nerve function deteriorated or new reaction symptoms developed, the trial treatment was stopped and an individually modified treatment was provided [15]. A decision tree was used to help clinicians decide whether or not patients should continue in the trial or receive individualized treatment in case of new NFI or reaction symptoms. Once individualized treatment was warranted, it was up to the clinician to decide on further patient management. The definition used to determine deteriorating MFT was: an increase of 6 points or more on the score per nerve since the last assessment, or an increase of 3 points or more on the score per nerve that was confirmed on the consecutive visit. VMT deterioration was defined as: a reduction in VMT score by two or more points or a reduction of 1 point on two consecutive visits. Removal from the trial treatment due to reactions was based on new or deteriorating reaction symptoms.

Patients who developed a serious adverse event (SAE) were removed from trial treatment and provided with individualized care. For both deteriorating and SAE cases, data collection continued up to the full 78 weeks.

### Primary and secondary outcomes

The primary study outcome was the proportion of patients with restored or improved nerve function (of all nerves) as measured by MFT and/or VMT at 78 weeks. Secondary outcomes considered six variables: individual nerves, impairment counts, RSS, SALSA-scale and P-scale scores between baseline and week 78, SAE’s between the intervention and control groups, and the proportion of patients needing additional prednisolone with considering differences in timing, dose and duration. Definitions for restoration, improvement and deterioration in these respective categories for secondary and primary outcomes were as previously described and are depicted in Table 1 [15].

**Table 1:** Overview of primary and secondary outcomes.

	Outcome	Definitions
Primary outcome	Proportion of patients with restored or improved nerve function	Using a composite score. Restored: all nerves back to normal function Improved: more restored* and/or improved** nerves than unchanged and/or deteriorated*** nerves Unchanged: same number of nerves impaired Deteriorated: more deteriorated nerves than improved and/or restored nerves
Secondary outcomes	1. Proportion of restored, improved, unchanged, deteriorated and fully impaired nerves for each specific nerve–e.g. ulnar nerve	For six sensory nerves and seven motor nerves
	2. Count of impairments	Per patient 26 nerves were assessed. Each impaired nerve adds 1 point to the total score
	3. Improvement Reaction Severity Scale	Improvement: reduction in RSS of 3 or more points on the total score, or a reduction of 2 or more points on any individual item of the scale
	4. Improvement in SALSA-scale and P-scale	Improvement: classified in a better category (extreme, severe, moderate, mild, none)
	5. Proportion of patients with Serious Adverse Events	
	6. Proportion of patients needing additional steroids for treatment of reaction or worsening NFI	

* restored: back to normal function

** improved: decrease of 3 points for MFT or increase of 1 point for VMT

***: deteriorated: increase of 3 points for MFT or decrease 1 point for VMT

### Data analysis

Data were entered at each centre in an Access Database and then combined to be analysed in Stata. Data were analysed according to the modified intention-to-treat principle: data of all randomized patients who matched the inclusion criteria were analysed for week 78, whether they had finished treatment or not, including all patients lost to follow-up. To handle missing data of patients lost to follow-up, the last observation carried forward method was used. For patients who received additional prednisolone, the assessment recorded at the time when additional prednisolone was first prescribed was carried forward. Only nerves with new impairments (<6 months) were included in the analyses. The primary and secondary outcomes were analysed using a Chi-Square test. A difference between treatment groups was considered significant when the p-value was < 0.05.

## Results

### Participants

**Fig 1:**
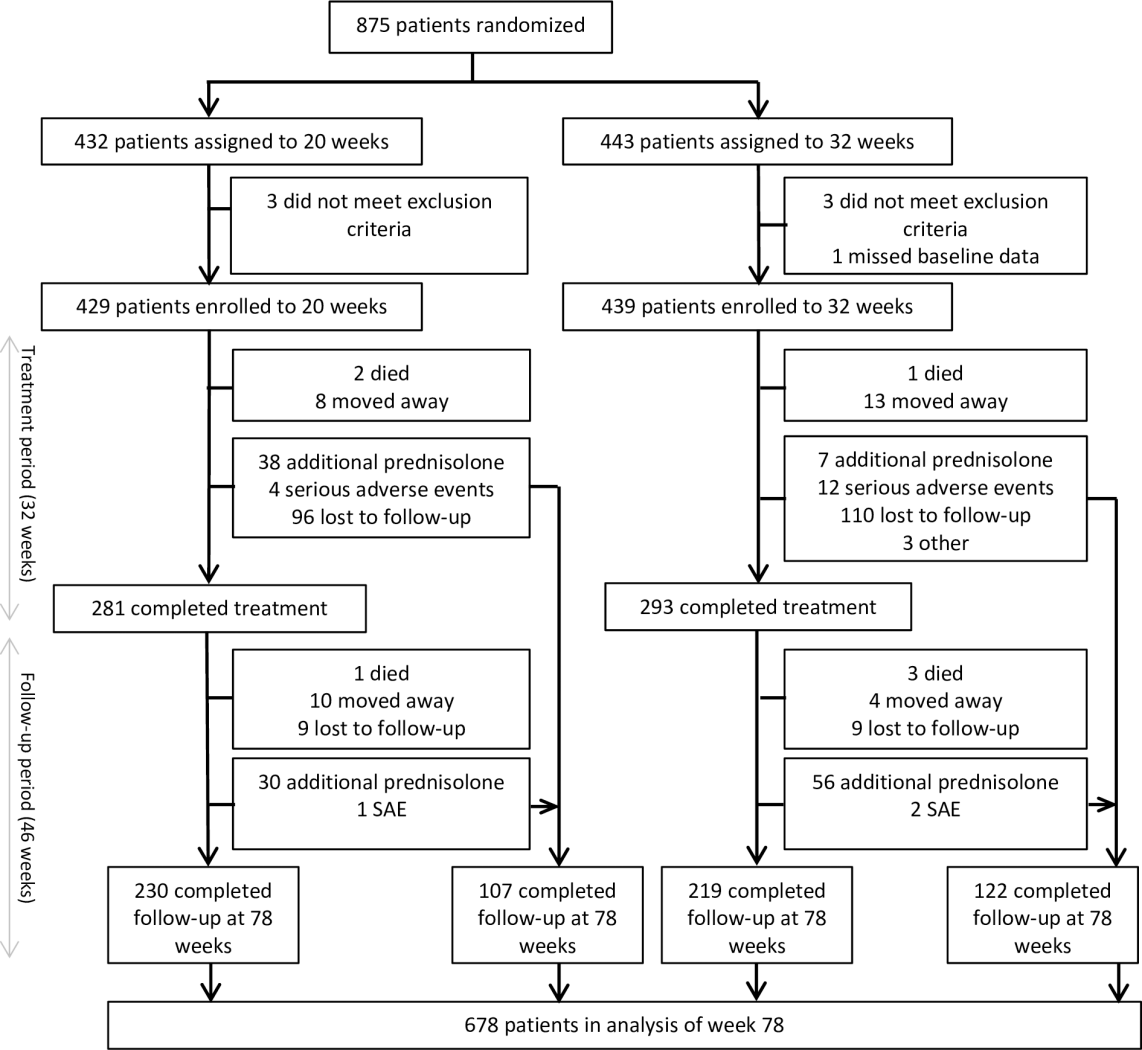
Flow diagram of number of patients enrolled, randomized, treated and followed-up.

A total of 875 leprosy patients were enrolled in the trial, of whom 432 were randomized to the 20-week arm and 443 to the 32-week arm. Patients were recruited between February 2012 and October 2013, and the last follow-up data were collected in July 2015. The flow diagram in Fig 1 illustrates the number of patients followed up and the reasons for drop out. Seven of the randomized patients were excluded in the final analyses: one patient who had missing baseline MFT assessments and six patients who did not meet the inclusion criteria of having recent NFI. The number included in the analyses reported here was therefore 868.

### Baseline data

**Fig 2:**
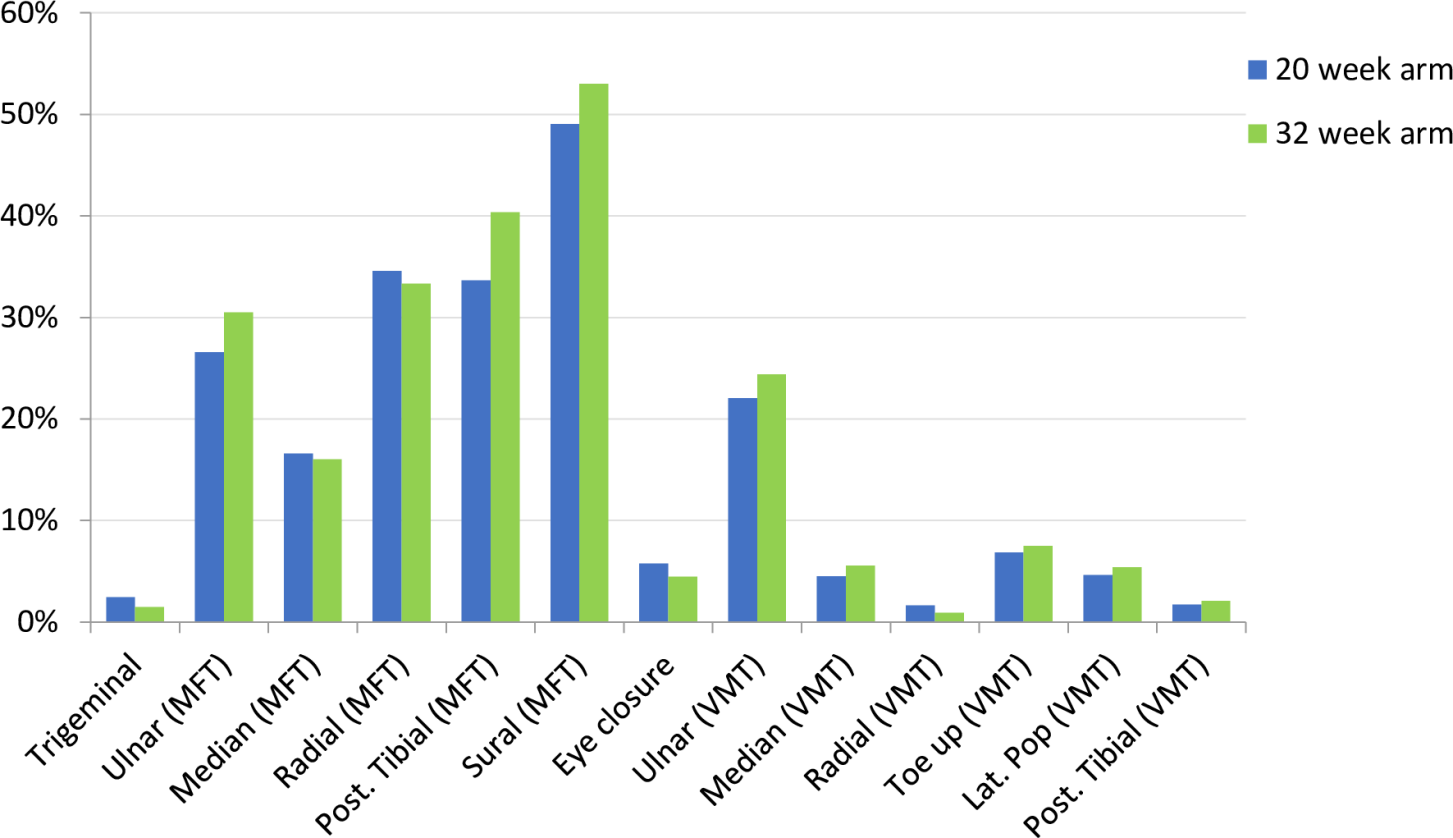
Baseline impairments for sensory and motor nerves.

The intake and distribution of several patient characteristics per research centre are presented in Table 2. Table 3 shows the baseline demographic and clinical characteristics of the total patient group. Differences in demographic and clinical characteristics did not reach statistical significance between the groups. At baseline, sensory function was more often impaired than motor function: for both groups the median number of nerves with impaired sensory function was 3, ranging from 0–12, while the median number of nerves with impaired motor function was 1 (0–13). The proportions of sensory and motor impairment per nerve are shown in Fig 2.

**Table 2:** Intake per research centre.

Centre	Number of patients	Gender (% female)	Mean age	Low weight group (%)	20-week arm (%)
Anandaban Hospital *Kathmandu*, *Nepal*	57	26%	36.7	37%	47%
Foundation for Medical Research *Mumbai*, *India*	82	15%	31.8	34%	49%
JALMA institute of Leprosy & Other Mycobacterial Diseases *Agra*, *India*	230	17%	31.5	53%	50%
Lalgadh Leprosy Hospital and Services Centre *Dhanusha*, *Nepal*	166	28%	34.3	45%	50%
Danish Bangladesh Leprosy Mission *Nilphamari*, *Bangladesh*	232	22%	39.1	51%	50%
Dr. Soetomo Hospital *Surabaya and Madura*, *Indonesia*	101	25%	31.9	46%	48%

**Table 3:** Baseline demographics and clinical characteristics of the 868 patients.

	20-week arm		32-week arm	
	(n = 429)		(n = 439)	
Gender (% female)	101	(23.4%)	90	(20.4%)
Age (mean ±SD)	34.7	(12.1)	34.3	(12.1)
Literate (%)	241	(56.5%)	247	(56.3%)
MB/PB (% MB)	339	(79.2%)	357	(81.0%)
RJ classification				
TT	9	(2.1%)	20	(4.6%)
BT	212	(49.4%)	203	(46.2%)
BB	61	(14.2%)	55	(12.5%)
BL	67	(15.6%)	70	(16.0%)
LL	40	(9.3%)	51	(11.6%)
PN	40	(9.3%)	40	(9.1%)
Average smear (mean ±SD)				
TT	0	(0)	0	(0)
BT	0.3	(0.8)	0.2	(0.8)
BB	0.3	(0.7)	0.6	(1.2)
BL	2.0	(1.5)	2.0	(1.7)
LL	3.0	(1.8)	3.2	(1.7)
PN	0.4	(0.8)	0	(0)
Disability grade				
0	171	(39.9%)	165	(37.6%)
1	158	(36.8%)	176	(40.1%)
2	100	(23.3%)	98	(22.3%)
T1R				
Mild	66	(15.4%)	59	(13.4%)
Severe	14	(3.3%)	24	(5.5%)
T2R				
Mild	11	(2.6%)	14	(3.2%)
Severe	2	(0.5%)	6	(1.4%)

MB: Multibacillary; PB: Paucibacillary; RJ: Ridley-Jopling; T1R: Type 1 Reaction; T2R: Type 2 Reaction

### Number analysed

Of the 868 patients enrolled in the study, the trial treatment period—the first 32 weeks—was completed by 281 (65.5%) in the 20-week arm and 293 (66.7%) in the 32-week arm. Complete follow-up data until week 78 were collected for 230 (82%) of those patients in the 20-week arm and 219 (75%) patients in the 32-week arm (see Fig 1). At week 78, follow-up data were collected for 229 additional patients who did not complete trial treatment due to new NFI, new or recurrent reactions, SAEs or loss to follow-up. For the intention-to-treat analyses, the primary and secondary outcomes were analysed using the data of all patients who met the inclusion criteria (n = 868). A separate per protocol analysis was carried out including only patients who had completed treatment (n = 574).

### Primary outcome

**Fig 3:**
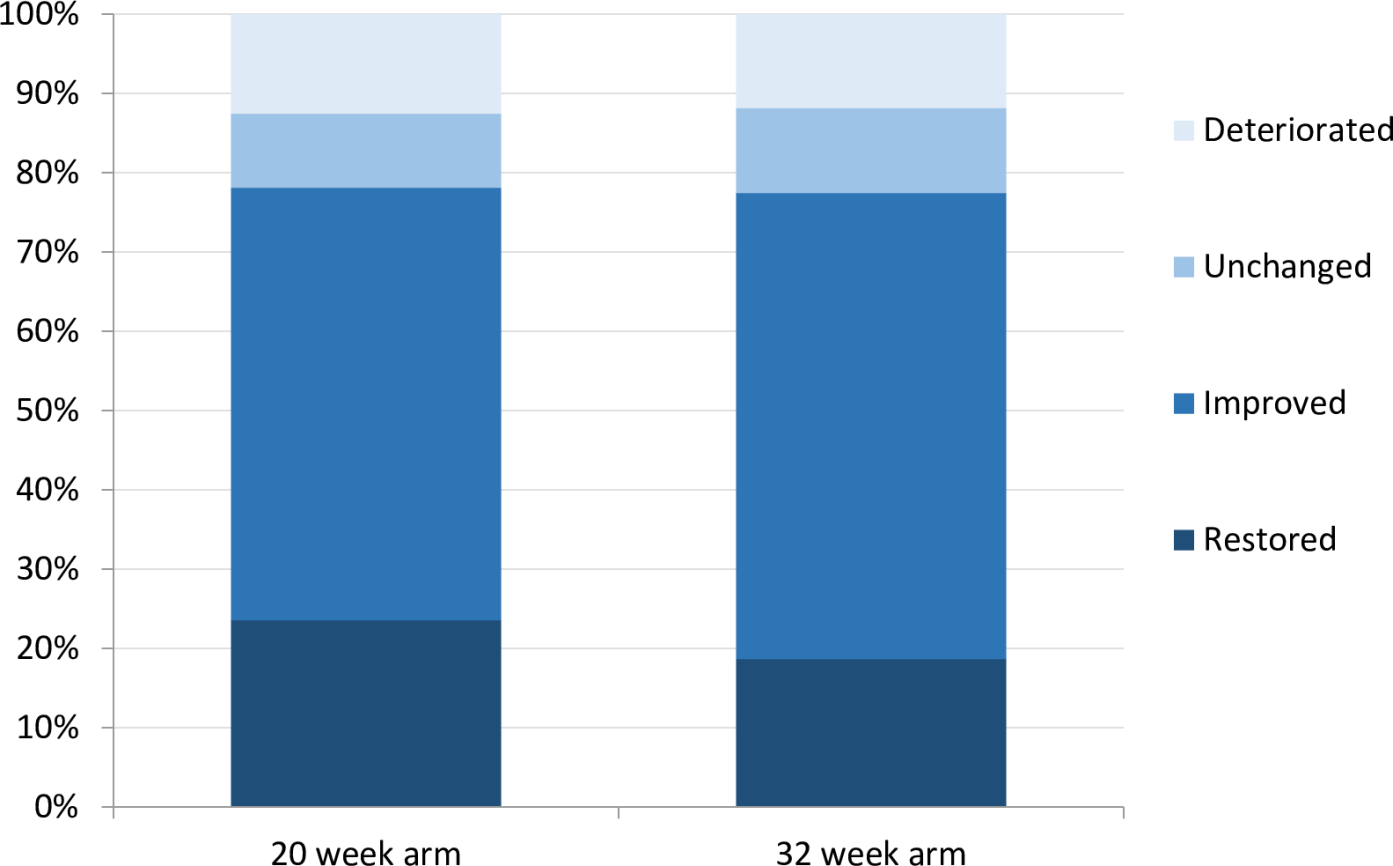
Percentage of patients with restored, improved, unchanged and deteriorated nerve function impairment at week 78 as primary outcome.

The proportion of patients with restored or improved nerve function at week 78 was almost similar in both groups: 78.1% in the 20-week arm and 77.5% in the 32-week arm (p = 0.821). At week 32 and week 52, this outcome was not significantly different between the two groups either. Fig 3 shows the proportion of patients in each category of improvement. In the 20-week arm, more patients showed completely restored nerve function than in the 32-week arm, 23.5% against 18.7%. The per-protocol analysis, leaving out treatment non-compliers, resulted in an overall slightly higher proportion of patients with restored and improved nerves. However, again no significant difference was found between the groups: 81.9% in the 20-week arm and 81.7% in the 32-week arm (p = 0.960).

### Secondary outcomes

Table 4 presents the outcomes per nerve, for six sensory and seven motor nerves. The proportion of restored and improved motor nerves was overall higher than for sensory nerves. The sensory function of the radial, sural and posterior tibial nerves had the highest proportion of improvement and restoration. The motor function of the ulnar and posterior tibial nerves improved most between baseline and week 78. The median count of impairments at baseline and at week 78 is shown in Table 5. 75.7% of the patients in the 20-week arm showed an improvement in count of impairment, compared to 70.8% in the 32-week arm (p = 0.149). Table 5 also presents the results of the Reaction Severity scale, the SALSA scale and the P-scale for every assessment point. The proportion of patients with improved in RSS score at week 78 was higher in the 32-week arm (70%) than in the 20-week arm (65%), but this difference was not statistically significant (p = 0.09). At 78 weeks, the day-to-day situation for patients had barely improved: the scores of both SALSA and P-scale reduced slightly between baseline and week 78. The change over time did not differ significantly between the two arms (p = 0.638 for SALSA and p = 0.543 for P-scale).

**Table 4:** Outcomes per nerve at week 78.

		n	Normal	Restored	Improved	Unchanged	Deteriorated
SENSORY			%	%	%	%	%
Trigeminal	20 weeks	858	97.4	1.5	-	0.9	0.1
	32 weeks	878	98.2	1.4	-	0.1	0.3
Ulnar	20 weeks	830	60.2	17.1	9.3	5.4	8.0
	32 weeks	850	55.3	17.8	11.9	5.7	9.4
Median	20 weeks	839	69.0	14.4	5.7	4.1	6.8
	32 weeks	860	67.1	12.2	8.7	4.0	8.0
Radial	20 weeks	814	49.6	24.1	11.4	6.0	8.9
	32 weeks	845	47.5	21.7	14.1	5.4	11.4
Posterior tibial*	20 weeks	810	51.4	17.0	11.0	9.9	10.7
	32 weeks	839	46.6	16.6	16.2	11.1	9.5
Sural*	20 weeks	784	41.5	14.8	17.6	14.2	12.0
	32 weeks	827	37.1	16.1	17.7	20.9	8.2
MOTOR		n	%	%	%	%	%
Eye closure	20 weeks	852	90.3	4.9	1.8	2.1	0.9
	32 weeks	871	91.9	4.8	1.5	1.0	0.8
Ulnar	20 weeks	826	61.0	15.3	7.1	10.4	6.2
	32 weeks	855	60.9	13.1	8.2	12.4	5.4
Median	20 weeks	850	84.5	8.1	0.6	2.7	4.1
	32 weeks	870	84.6	8.3	1.3	3.2	2.6
Radial	20 weeks	856	95.7	3.0	0.1	0.2	0.9
	32 weeks	877	95.4	2.9	0.1	0.6	1.0
Lateral popliteal (dorsiflexion foot)	20 weeks	850	90.5	5.9	1.1	1.7	0.9
	32 weeks	871	89.8	5.9	0.8	2.3	1.3
Posterior tibial up	20 weeks	833	79.4	10.8	2.2	4.6	3.1
	32 weeks	861	81.2	9.1	1.7	5.7	2.3
Posterior tibial down	20 weeks	826	77.2	11.6	0.4	5.8	5.0
	32 weeks	860	76.5	12.9	0.4	6.9	3.4

*Difference between arms is statistically significant

**Table 5:** Secondary outcomes: impairment, reaction severity scale, SALSA scale, P-scale.

	20-week arm		32-week arm		
	n	median	n	median	Test statistic
		(IQ range)		(IQ range)	
**Count of impairments**					
Baseline sensory	429	3 (1–5)	439	3 (1–6)	NS
Baseline motor	429	1 (1–3)	439	1 (1–3)	NS
Total count baseline	429	4 (2–8)	439	4 (2–8)	NS
Week 78 sensory	338	1 (0–4)	343	2 (0–4)	NS
Week 78 motor	338	0 (0–2)	343	1 (0–2)	P<0.05
Total count week 78	338	2 (0–5)	343	2 (1–6)	NS
Difference total count baseline- week 78	338	2 (0–4)	343	2 (0–4)	NS
**Reaction Severity Scale**					
Baseline	426	4.5 (2–8.5)	438	5.5 (2.5–9.5)	NS
Week 32	425	2 (0–5.5)	437	2.5 (0–6)	NS
Week 52	427	2 (0–5.5)	437	2.5 (0–6)	NS
Week 78	422	2 (0–5.5)	432	2 (0–6)	NS
Difference total count baseline- week 78	420	2.5 (0–5)	432	3.5 (0–6)	NS
**SALSA scale**					
Baseline	402	23 (20–35)	412	24 (20–35)	NS
Week 78	269	20 (20–24)	272	20.5 (20–26)	NS
Difference baseline- week 78	269	0 (-1–3)	272	0 (-1–3)	NS
**P-Scale**					
Baseline	402	12.5 (1–21)	412	9 (0–20)	NS
Week 78	269	5 (0–18)	273	6 (0–18)	NS
Difference baseline- week 78	269	0 (-16–7)	273	0 (-18–5)	NS

IQ: inter-quartile; NS: not significant

**Fig 4:**
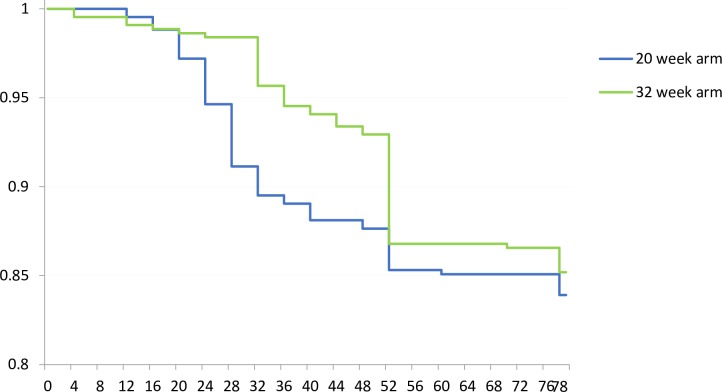
Time until first event requiring additional prednisolone, i.e. a leprosy reaction and/or new NFI and/or deteriorating NFI (n = 868).

Additional prednisolone, to treat new or deteriorating NFI and reactions, was required in 68 (16%) patients in the 20-week arm and 65 (15%) patients in the 32-week arm. This difference was not statistically significant. Interestingly, the majority (38/68) of these patients in the 20-week arm needed additional prednisolone between week 21 and week 32, while in the 32-week arm additional prednisolone was given mostly between week 33 and 52 (56/65). In the 32-week arm, the additional prednisolone given before week 32 was largely (78%) due to reaction—with or without accompanying worsening of NFI, while the additional prednisolone given after week 32 was more often for deteriorating NFI without reaction (55% of the cases needing additional prednisone did not have other reaction symptoms). Fig 4 shows the time until the first event requiring an additional prednisolone. The dose and duration of additional prednisolone treatment did not significantly differ between the two groups.

When zooming in on the group of patients that needed additional prednisolone (independent of treatment arm), it became apparent that their baseline characteristics differed from the group that did not need additional prednisolone. Table 6 gives an overview of these characteristics. Next to the presented variables, patients needing additional prednisolone during or after the trial period also showed significantly more often other reaction signs, i.e. inflamed and raised skin lesions, peripheral oedema, nerve pain and tenderness.

**Table 6:** Overview of differences in baseline characteristics between the group that needed additional prednisolone and the group that did not need additional prednisolone.

		Additional prednisolone		No additional prednisolone		p-value
		(n = 134)		(n = 734)		
**WHO classification**	% MB	120	(89.6%)	576	(78.5%)	0.003
**Ridley-Jopling**						<0.001
**classification**	TT	2	(1.5%)	27	(3.7%)	
	BT	61	(45.5%)	354	(48.2%)	
	BB	10	(7.5%)	106	(14.4%)	
	BL	32	(23.9%)	105	(14.3%)	
	LL	23	(17.2%)	68	(9.3%)	
	PN	6	(4.5%)	74	(10.1%)	
**Skin smear (BI)**	Mean	1.4		0.7		<0.001
	Positive	60	(44.8%)	157	(21.4%)	<0.001
**Eye-hand-foot score**	Mean		1.9		1.5	0.014
	Positive	94	(70.2%)	438	(59.7%)	0.022
**Type 1 reaction**	Positive	44	(32.8%)	119	(16.2%)	<0.001
**Type 2 reaction**	Positive	10	(7.2%)	22	(3.0%)	0.011
**Weight group**	% high weight	82	(61.2%)	375	(51.1%)	0.031
**BMI**	Mean	20.4		19.8		0.024

### Adverse events

There were seven deaths, three in the 20-week arm and four in the 32-week arm; but none were related to trial treatment. SAE occurred in both groups, but were more often reported in the intervention group: 12 cases (2.7%) in the 32-week arm and four cases (0.9%) in the 20-week arm [21]. This difference was statistically significant (p = 0.04). The main reported SAE’s were hypertension (6), diabetes (6), and peptic ulcer (2): all commonly recognized side effects associated with prednisolone treatment. Minor side effects were reported in 66% in the 32-week arm and 68% in the 20-week arm. Detailed results on adverse events will be reported separately.

### Additional analyses

We used logistic regression to assess the (univariate) association between clinical and demographic characteristics recorded at baseline and the primary outcome of improved or restored nerve function. The five characteristics that demonstrated a significant correlation with the primary outcome were weight group, body mass index (BMI), gender, WHO disability grade and Eye Hand Foot (EHF) score. A patient with a higher BMI had higher odds on improved or restored nerve function at 78 weeks (OR = 1.09 (1.04–1.14)). The high weight group (>50 kg) had a 1.43 (1.09–1.86) higher probability of a positive primary outcome than the low weight group. Furthermore, women were 1.46 (1.06–2.10) times more likely to have a restored or improved nerve function than men. The subgroup analysis also demonstrated that patients having a grade 1 or 2 impairment at baseline were less likely to have restored or improved nerve function at week 78 (OR Grade 1: 0.73 (0.54–0.98) and OR Grade 2: 0.19 (0.12–0.28)). A higher EHF score also reduced the odds of a good outcome (OR: 0.73 (0.67–0.80). The following baseline characteristics had no influence on improved nerve function: WHO classification, Ridley-Jopling classification, BI (skin smear) and the presence or absence of severe Type 1 or Type 2 reaction.

Using a Cox Proportional Hazard model, we assessed whether there was a trend across the entire duration of follow up regarding primary outcome -i.e. not limited to 32, 52 and 78 weeks. The conclusion from this analysis was similar to what has been described above, there was no effect of treatment arm on primary outcome.

## Discussion

In both treatment arms in our study, a large majority (78%) of patients showed improvement and restoration of nerve function, indicating that 20-week prednisolone treatment is sufficient for most patients. However, around 15% of the patients required individualized further treatment because of reactions or deteriorating NFI. Notably, the proportion of patients needing additional prednisolone was similar in both groups (15% and 16%), and the duration and dose of the additional prednisolone treatment did not differ either. However, we did see a difference in timing of the need for additional prednisolone. New NFI and reactions were primarily reported in the first few weeks after prednisolone treatment had ended, thus after 20 weeks and 32 weeks respectively for the control and intervention arm. A similar rebound effect was observed in the Trials in Prevention of Disability (TRIPOD) study and in the study of Rao et al. [14,22]. The longer prednisolone treatment in our study seems to merely postpone the immune response in some patients.

The roughly 15% of patients that required additional prednisolone differed at baseline from the group that did not need extra prednisolone. This former group had significantly more MB patients, mainly BL and LL, higher average skin smear, higher EHF score, had T1R and T2R more often at baseline and showed more often increased signs of reaction, i.e. inflamed and raised skin lesions, peripheral oedema, nerve pain and tenderness. Further studies of an even longer prednisolone course than assessed in this study might be beneficial for these higher risk patients, as it seems that the immune response against dead *M*. *leprae* bacilli is too persistent to be permanently suppressed by a 32-week prednisolone course [23,24]. At present, however, our results reinforce that 20 weeks tapered prednisolone with an extended higher dose plateau is sufficient treatment for the majority of recent leprosy neuropathy cases, while extended individualized treatment will be needed for some patients, which may be associated with commonly recognized risk factors for complications.

Women overall had a better response to prednisolone then men. This was unexpected as previous studies show that women generally have longer delay in presenting with leprosy and may then have more severe NFI at time of diagnosis with less chance for recovery [25–27]. Though, in our study we only included patients with a delay of less than 6 months and therefore a pre-selection of women with recent NFI was made.

The group of patients needing additional prednisolone had significantly more often certain clinical baseline characteristics that are frequently associated with reduced outcomes, such as high BI, MB classification and reactions. When analysing the correlation between baseline characteristics and the primary outcome, i.e. improved/recovered vs. unchanged/deteriorated NFI, we had expected that patients with deteriorated NFI would also have significantly more often a high BI, MB classification and reactions at baseline. This was not the case, however. One explanation may be that the group of ‘unchanged’ patients diluted the group of patients with a deterioration, and made the association between these specific baseline characteristics and primary outcome less strong.

The mechanisms behind nerve damage in leprosy are generally not well understood and challenging to study. Nerve biopsies can give some insight in the pathophysiology of neuropathy, but are limited to a single point in time and do not provide information about the onset or further development of impairment [23]. Several mechanisms are described in literature though. In vitro, demyelination has been shown to occur when *M*. *leprae* invades the Schwann cells [28–30]. This process can take place in an early stage, when the immune system has not been activated yet. Secondary immune responses likely play a role via inflammatory cytokines [31–34] and T-cells [35–38], leading to demyelination or damaging of Schwann cells. Last, mechanical effects such as oedema and ischemia [23,39] can lead to axonal loss. Nerve regeneration can occur after the inflammation is controlled; however, the duration of the inflammation and nerve scarring may influence outcome [7, 39]. As a strong immunomodulator, prednisolone can impact secondary responses to reduce the inflammation, reducing oedema and scar formation [7]; thereby, providing time and space for nerve regeneration to occur if applied within <6 months of symptoms [2,8,40, 41]. When the delay in prednisolone treatment is too long (>6 months), the damage to the nerve is considered to be irreversible. From previous studies [5,42], it seems that NFI of shorter onset duration prior to treatment has better chances on improvement.

Observational studies in leprosy neuropathy have shown that prednisolone improves nerve function [2,10–12]. Although prednisolone is generally considered an important drug for improving NFI, it is not known by what mechanisms or to what extent prednisolone is responsible for the reported improvements, such as in placebo controlled randomized controlled trials like the TRIPOD studies [22,43]. Several studies have also indicated that a high proportion of patients (33–75%) experience spontaneous nerve function improvement even if left untreated, depending on the severity of NFI and type of nerve [22,44–46]. Even in 51% of placebo patients with old NFI (> 6 months of symptoms) improvement can be demonstrated without prednisolone treatment [45]. A placebo-controlled RCT could be an important next step to investigate the actual effect of prednisolone on the treatment of recent NFI. Alternatives for prednisolone should continue to be sought, as so far no treatments have been proven effective. For example, azathioprine and cyclosporine, immunosuppressants used to treat reactions, show only limited effect in studies inclusive of but not specifically for NFI [46–48]. Nerve decompression, a surgical method to relieve severe inflammatory pressure, is sometimes used to improve ongoing neuropathy in leprosy; however, controlled randomized trial evidence on the effectiveness of this method is lacking as well [49].

The strongest point of our study is the multi-centre, multi-country design which enabled representative sampling across leprosy demographics in South East Asia, where the bulk of global leprosy cases are diagnosed. This RCT was unique in maintaining a middle plateau for a longer time with a relatively high dose of prednisolone and demonstrated that a 20-week treatment was sufficient for the majority of recent neuropathy cases. This RCT was also able to demonstrate that baseline BMI of the patient was directly related to outcomes. This highlights the practical and clinical relevance for contextual realities, such as extreme poverty and malnutrition present within some leprosy-affected populations [50–52]. Such issues can represent individual complications for health and healing during a neuropathy episode.

At the conclusion of the study, three limitations became apparent. At the 78-week time point, only 72% of the patients in the 20-week arm and 78% of the patients in the 32-week arm provided data, exceeding the Evidence Based Medicine cut off of 20%, leading to a loss of validity [53]. In addition, treatment data was absent at other time points due to SAE, the need for additional prednisolone or temporary loss-to-follow up. To limit potential bias related to non-random loss of patients, data was analysed according to the intention to treat principle, using the last observation carried forward approach. By using this approach, the results of this study are likely conservative with the actual improving effect of prednisolone on NFI possibly even larger. A second limitation is that the duration of NFI prior to intake was self-reported, which may have allowed recruitment of patients with previous symptoms beyond the 6 month cut-off for inclusion and who were, therefore, less likely to demonstrate nerve improvement with treatment [5,41]. The third limitation is that Ridley-Jopling classifications were based on clinical aspects only, except for one centre that performed histopathology. The results of the logistic regression to evaluate the effect of RJ classification on the final result should therefore be interpreted with care.

We conclude that a 20-week course is sufficient to improve nerve function in 78% of patients with recent NFI. Future studies should focus on improved regimens or alternative treatments, as approximately 15% of patients with recent NFI require additional prednisolone. Moreover, alternative therapies could potentially reduce risks for developing steroid-dependency and other long term treatment side effects. Secondly, it is important to better unravel the pathophysiology of nerve function impairment and to study the actual effect of prednisolone on immune function as it relates to NFI. Understanding the mechanisms behind NFI could lead to alternative, more effective solutions for the treatment of NFI and the prevention of irreversible impairments and subsequent disabilities.

## Supporting information

S1 Data Dataset TENLEP clinical trial. (XLSX)
